# Healthcare worker perceived barriers and facilitators to implementing a tuberculosis preventive therapy program in rural South Africa: a content analysis using the consolidated framework for implementation research

**DOI:** 10.1186/s43058-023-00490-8

**Published:** 2023-08-30

**Authors:** Brittney J. van de Water, Michael Wilson, Karl le Roux, Ben Gaunt, Sarah Gimbel, Norma C. Ware

**Affiliations:** 1 Boston College, Connell School of Nursing, Chestnut Hill, MA USA; 2 Department of Health Behavior, University of North Carolina Gillings School of Global Public Health, Chapel Hill, NC USA; 3 Advance Access and Delivery, Durban, South Africa; 4 University of Cape Town, Cape Town, South Africa; 5 Family Medicine Department, Walter Sisulu University, Mthatha, South Africa; 6 Zithulele Research and Training Centre, Giniytasambi, South Africa; 7 Eastern Cape Department of Health, Bhisho, South Africa; 8 Department of Child, Family & Population Health Nursing, University of Washington, Seattle, USA; 9 Department Global Health and Social Medicine, Harvard Medical School, Boston, MA USA

**Keywords:** Content analysis, Tuberculosis, Health system strengthening, Preventive therapy, Implementation science, Nursing, Consolidated Framework for Implementation Research (CFIR), Knowledge, Attitudes, Beliefs

## Abstract

**Background:**

South African national tuberculosis (TB) guidelines, in accordance with the World Health Organization, recommend conducting routine household TB contact investigation with provision of TB preventive therapy (TPT) for those who qualify. However, implementation of TPT has been suboptimal in rural South Africa. We sought to identify barriers and facilitators to TB contact investigations and TPT management in rural Eastern Cape, South Africa, to inform the development of an implementation strategy to launch a comprehensive TB program.

**Methods:**

We collected qualitative data through individual semi-structured interviews with 19 healthcare workers at a district hospital and four surrounding primary-care clinics referring to the hospital. The consolidated framework for implementation research (CFIR) was used to develop interview questions as well as guide deductive content analysis to determine potential drivers of implementation success or failure.

**Results:**

A total of 19 healthcare workers were interviewed. Identified common barriers included lack of provider knowledge regarding efficacy of TPT, lack of TPT documentation workflows for clinicians, and widespread community resource constraints. Facilitators identified included healthcare workers high interest to learn more about the effectiveness of TPT, interest in problem-solving logistical barriers in provision of comprehensive TB care (including TPT), and desire for clinic and nurse-led TB prevention efforts.

**Conclusion:**

The use of the CFIR, a validated implementation determinants framework, provided a systematic approach to identify barriers and facilitators to TB household contact investigation, specifically the provision and management of TPT in this rural, high TB burden setting. Specific resources—time, trainings, and evidence—are necessary to ensure healthcare providers feel knowledgeable and competent about TPT prior to prescribing it more broadly. Tangible resources such as improved data systems coupled with political coordination and funding for TPT programming are essential for sustainability.

**Supplementary Information:**

The online version contains supplementary material available at 10.1186/s43058-023-00490-8.

Contributions to the literature

- The CFIR provides a systematic approach to identifying barriers and facilitators for healthcare workers to TB prevention programming in a rural, Low resource setting, prior to implementation.
- Integrating TB prevention education throughout nursing and medical education and continuing education could support healthcare workers feel more knowledgeable about TPT and support them in prescribing it more broadly, especially as it is scaled-up in South Africa with recently expanded guidelines.
- Providing healthcare workers with tangible resources such algorithms at the point-of-care and simple documentation systems may improve the success and sustainability of a comprehensive TB program.

## Introduction

To provide tuberculosis preventive therapy (TPT) to high-risk individuals, health systems—and healthcare workers (HCWs)—must effectively screen individuals to rule out disease and, if appropriate, initiate TPT. Systematically screening individuals from high-risk groups is often challenging in low-resource settings. However, once disease is ruled out, TPT is 60–90% effective [1–4].

Currently, little is known about HCWs knowledge, attitudes, and beliefs regarding TPT to asymptomatic household contacts in low-resource, high TB incidence settings, such as South Africa [5, 6]. Perceptions of HCWs implementing interventions is critical to its success, especially in South Africa where the landscape of TPT guidelines and regimens are quickly evolving [7, 8]. Poor HCW adoption leads to limited maintenance of interventions, making interventions more likely to fail [9]. Therefore, prior to implementing a comprehensive TB program in rural South Africa, the aim of this study was to understand HCWs’ perceived barriers and facilitators on factors influencing TPT provision and drivers of implementation success or failure using the Consolidated Framework for Implementation Science (CFIR).

## Methods

### Setting

**Fig. 1:**
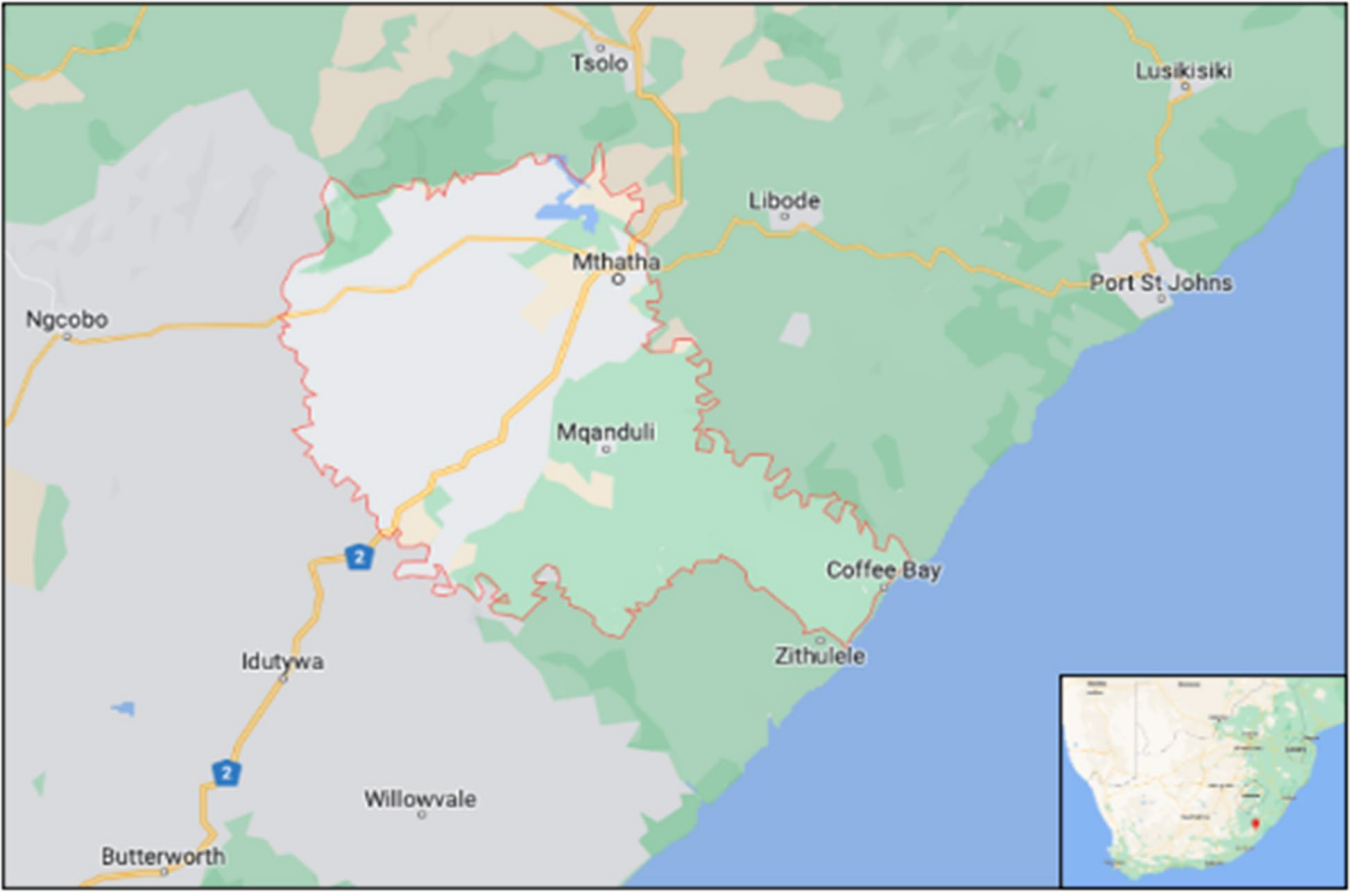
King Sabata Dalindyebo District Municipality

This study took place in the King Sabata Dalindyebo (KSD) sub-district, Eastern Cape (Fig. 1). The district hospital serves nearly 130,000 people and supports surrounding clinics. Since 2014, TPT has been routinely recommended for people living with HIV and children under five who are household contacts of patients with TB per South African policy [10].

### Study design

This was a baseline qualitative study inform the implementation of a forthcoming, comprehensive TB program. The original CFIR was used to guide interview development, prior to dissemination of the updated 2022 CFIR 2.0 [11]. Standards for Reporting Qualitative Research (SRQR) was used to adhere to reporting guidelines [12].

### Sampling and recruitment

Purposive sampling was used to recruit HCWs directly involved with the TB program. Participants included doctors, professional and enrolled nurses, radiographers, pharmacists/pharmacy technicians, and TB data managers. All participants were over 18 years (Table 1).

**Table 1:** Description of participants

Interview #	Place	Type of interview	Educational background
1	H	Hospital MO	Male, 30s; < 5 years at this hospital
2	C	Clinic OM	Female, 40s; professional nurse > 10 years, OM for 1 year
3	H	Hospital MO	Male, 30s; 5 years at this hospital
4	H	Hospital MO	Male, 40s; 15 years at this hospital
5	H	Data capturer	Male, 20s; 2 years as data clerk at this hospital/catchment area
6	C	Clinic OM	Female, 40s; professional nurse for 25 years; OM for 1 year
7	H	Pharmacist	Female, 30s; at this hospital for 3 years
8	C	Clinic OM	Female, 40s; professional nurse; 6 years at this clinic
9 + 10	C	Clinic ENs	Female, 30s; enrolled nurse; 1 year in KSD; 10 years as CHW now EN after 2 years of nursing school Female, 30s; enrolled nurse ^b^Two interviewees
11	C	Clinic pharmacy assistant	Pharmacy assistant since 2019^a^
12 + 13	H	Radiographers	Male, 30s; 1 year at this hospital Female, 30s; 5 years at this hospital^b^
14	C	Clinic nurse	Professional nurse for 6 years^a^
15	H	Hospital MO	Female, 30s; MO for 5 years. Family Medicine registrar
16	H	Hospital MO	Female, 30s; MO for 15 years
17	C	Clinic OM	Professional nurse for 21 years, OM at this clinic 14 years^a^
18	H	Hospital MO	Male; 30s; at this hospital for 7 years
19	H	Hospital TB nurse	Female, 30s; professional nurse for 4 years

*H*, hospital based; *C*, clinic based; *MO*, medical officer; *OM*, office manager

^a^Gender and age not recorded

^b^Two interviewees

### Data collection

All interviews took place at the hospital and four referral clinics in April 2021. Data were collected, confidentially in private offices, via individual semi-structured interviews lasting 30 to 60 min. Interview guide included (Supplement 1). Interviews were conducted in English and audio-recorded. Written informed consent was obtained prior to participation. No compensation was provided. Transcription was completed by Lain Transcription and uploaded into Dedoose for coding [13].

### Data analysis

**Fig. 2:**
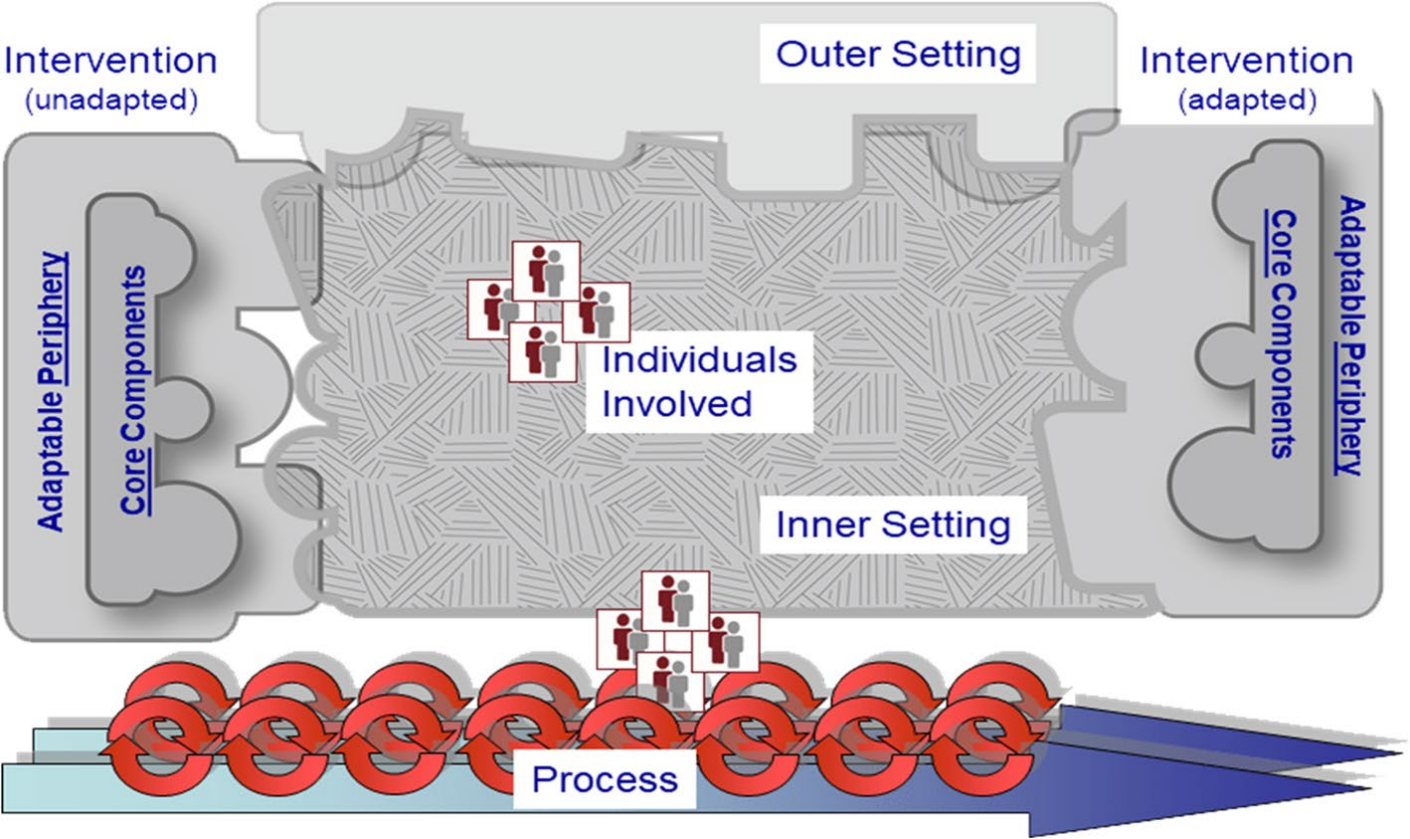
Consolidated framework for implementation research (copyright: CFIR)

A deductive content analytic approach was used to code descriptive quotes identified through analysis. The CFIR codebook, including pre-specified domains and constructs, was used to guide analysis. Illustrative quotes were subsequently mapped onto the CFIR framework (Fig. 2). Data were reviewed and coded by BvdW and MW. To ensure inter-coder reliability, 20% (*n* = 4) of interviews were double coded. Coders discussed discrepancies until consensus was achieved and coders agreed on to which construct a code (or quote) mapped. Subsequent interviews were coded independently. Coders went through codes, by construct, synthesizing barriers and facilitators to implementation (Tables 2 and 3).

**Table 2:** Barriers to implementing TB preventive therapy into a comprehensive TB program in a rural setting by CFIR domain and construct

Barriers	Construct
**Intervention characteristics**	
Formal training of HCWs	Knowledge, evidence strength
Knowledge of government pharmaceutical procurement protocols and schedules	Complexity/access to knowledge and information
Lab processing time, patients have to wait overnight at hospital for results	Complexity
**Outer setting**	
Transportation	Needs of community
Time and money to get to clinics	Needs of community
Education regarding TPT	Needs of community
Increased pill burden	Needs of community/patients
Not feeling sick, so why take medicine	Patient needs and resources
**Inner setting**	
Majority of community HCW’s are based in clinics and not in community	Readiness for implementation
Stock outs of medication	Readiness for implementation
Concerns around regimens, toxicity, and prescribing	Readiness for implementation/access to knowledge and information
Ongoing debate/lack of consensus about IPT among healthcare providers	Readiness for implementation/relative priority
Network/connectivity not always reliable at clinics, challenging to do online trainings, upload data, etc	Readiness for implementation/available resources
Competing prioritization of TPT in clinics and hospitals	Priorities, culture
Community stigma associated with TB and HIV	Compatibility
**Characteristics of individuals**	
Limited training of healthcare workers on clinical assessment, may contribute to missing cases	Knowledge and beliefs about the intervention
Challenging access to the community because of poor roads and infrastructure	Complexity/readiness for implementation
Communities are not taking DS-TB as seriously compared with HIV or DR-TB	Knowledge and beliefs about the intervention
Flexibility in health provider’s visit/agenda with patients	Implementation climate/relative priority
Fear of making a mistake or a misdiagnosis	Self-efficacy/knowledge of intervention

**Table 3:** Facilitators to implementing TB preventive therapy into a comprehensive TB program in a rural setting by CFIR domain and construct

Facilitators	Construct
**Intervention characteristics**	
Start with people who are easy to access and who should already be getting TPT—including people who are living with HIV and pregnant women	Design quality and packaging
De-centralized medication collection points such as the Central Chronic Medicines Dispensing and Distribution (CCMDD) models	Design quality and packing/networks
Utilizing existing medication pre-packaging programs to decrease congestion at hospitals and clinics	Design quality
**Inner setting**	
Clinics are enthusiastic to engage and follow guidelines	Readiness for implementation
Dedicated nursing staff exist to focus on TB at the hospital	Readiness for implementation
WhatsApp groups and other communication channels among clinical teams already exist to communicate about stock supply, scheduling, patient linkage, etc	Implementation readiness/networks and communications
Access to communities and households via existing ward-based outreach teams and CHWs	Implementation readiness, available resources
Existing triage processes within clinics where individuals are supposed to be screened for TB and asked about contacts	Readiness for implementation
Existing journal clubs at hospital to discuss guidelines, implementation, and evidence	Access to knowledge
Existing partnerships between the clinics and hospital; including medical officers visiting clinics regularly for complex patients	Networks and communication
**Process**	
Household champions are easy to identify in the community (i.e., grandmothers)	Champions
The ability of identified community members, including chiefs to organize and inform the masses	Champions

### Ethics approval

Ethics approval was obtained from Harvard Medical School, Boston, MA (IRB20-2122), Walter Sisulu University, Mthatha, South Africa (014/2021), and Eastern Cape Department of Health (EC_202104_002).

## Results

Nineteen participants were interviewed. Interviewees included ten hospital- and nine clinic-based individuals with a total of eight nurses, six doctors, two radiographers, two pharmacists, and one TB data clerk (Table 1). Ages ranged from 20 years to mid-50 s.

## Results by CFIR domains and constructs

### Intervention characteristics

#### Design quality and packaging of the intervention: looking at the “big picture” and patient-centered design

Participants described many topics around design quality and packaging the program well, specifically having innovative, novel interventions reflecting the needs of patients and providers. Quality documentation for TPT also arose. Currently, there are no systems in place to ensure follow-up and treatment for individuals on TPT (Quote Line Number 38, Supplement 2). Conceptualizing a comprehensive data management system that remains simple and algorithmic is important to overall design of the intervention. This system could be incrementally introduced, until the program is functioning at full capacity (Line 49, 52, 54, 55).

Participants also discussed the need for interventions to be patient-centered. First, to increase adherence, some HCWs recommended providing patients with the full length of treatment upon initiation rather than monthly refills (Line 50). Also, having the intervention clinic-based rather than hospital-based was suggested as improving success. This would improve feasibility and sustainability as clinics are embedded within communities, closer to patients’ homes, and provide ongoing patient management (Line 63).

#### Complexity: improving capacity at the clinic and community level

Similar to design quality and packaging, the complexity of providing TPT and reaching patients at the household-level came up often. For example, access to household contacts is difficult because the burden rests on patients (passive case finding) or strains the health system (Line 13). Once household contacts are identified, having a sensitive *and* specific algorithm for ruling out TB disease among different populations complicates screening and examinations of contacts. Participants felt it was important to understand who should do certain tasks (i.e., community health workers, nurses, doctors) for program success. Once patients initiate TPT, multifaceted challenges remain. In particular, ensuring patients stay engaged with care and adhere to TPT can be difficult (Line 16). The duration of the intervention (i.e., following each family for at least 12 months) and deciding which clinics and patients to prioritize were additional complexities discussed (Line 26).

### Outer setting

#### Needs and resources of those served by the organization: high community needs and few resources

Many participants discussed the significant needs of the community pertaining to this TB program (Line 166). Some HCWs described the importance of providing community-based education via a TPT campaign ensuring community members understand what TPT is and why it is important (Line 165). Lack of transportation and prohibitive costs of transport were consistently mentioned (167, 168, 169). Additionally, getting TB-exposed community members to clinics was perceived as difficult for many HCWs (Line 174, 176).

Another theme within this domain was the discussion of perceived acceptability of patient engagement. For example, participants felt adolescents and men were hard groups to reach; however, elderly individuals may be easier to engage. Therefore, elders could be enablers and support other family members (Line 177). Other participants felt women and children would be easier to reach than men for TPT (Line 181).

Documentation for TPT was also noted as a resource barrier at both hospital and clinics (Line 183). Another barrier to success were stock outs of medications, particularly in clinics, most often reported by doctors and pharmacists (Line 186).

### Inner setting

#### Readiness for implementation—available resources: nurses and community health workers need additional support and resources

Concepts that came up regarding the hospital and district’s readiness for implementation specific to available resources varied from easy to complex, including the need for better access to diagnostics. For example, one doctor said “Unfortunately, we don’t have 24-h lab services. So, if you see a patient after hours and you’re concerned about TB, unfortunately they'll have to sleep over [in the outpatient department] with other patients…which is quite a risk, but we can’t send them home because there's no transport and it’s quite far out for most of our patients” (Doctor, Male, Line 212).

Many HCWs discussed the need for resources to aid clinicians ruling out TB disease and prioritizing who receives TPT. Additionally, medical charts to document TPT prescriptions are absent. This lack of documentation infrastructure has been a reason why some HCWs believe TPT has never been prioritized or implemented effectively. The suggestion of algorithms for clinician utilization came up again (Line 218, 215, 219).

#### Implementation climate—relative priority: survival mode versus sustained momentum

Individuals described TB screening programs often lose priority because of the day-to-day “fires” many clinicians metaphorically put out (Line 98, 106, 108). Participants mentioned they often function in “survival mode” and adding another program may be too much (Line 99). A TPT program is seen as important until something like COVID comes along and derails momentum (Line 100). Also, some participants noted screening for TB can be overwhelming because it is too big of a problem to tackle (Line 104).

Additionally, the need to shift HCWs perspective on preventing drug-sensitive TB was a theme among some respondents. HIV is taken seriously and seen as a disease to prevent and treat, while TB is less clear. However, some HCWs viewed screening and close follow-up for TPT as feasible and especially urgent for drug-resistant TB (Line 112, 114).

Nevertheless, for the apathy some participants reported, multiple participants brought up the central role nurses should play in TPT delivery. Nurses were thought to be better suited to screen patients for TB than doctors (Line 83) and nurses could improve TB care through TPT if they were motivated, provided with appropriate training, had support from colleagues, and given time to provide patient education (Lines 234, 236, 238).

#### Readiness for implementation—access to knowledge and information: improved access to information is desperately needed

Access to knowledge and information about TPT and how to incorporate it into daily workflows was lacking for many HCWs. They discussed rarely learning about TPT in university and feeling unprepared to teach others about TPT when they had poor understanding themselves (Line 199, 198, 200). A pharmacist suggested how an online platform (Knowledge Hub) was used to quickly disseminate information about COVID-19, and a similar, platform could allow clinicians to access information about TPT too.

#### Implementation climate—learning climate: optimism about the future of TB care

The learning climate was one construct where optimism was most present. Many participants described their lack of training in TPT management, yet even with little previous training, there was great willingness to learn about TPT and the recognition for the continuous nature of learning needs (Line 89). Time constraints were palpable among clinicians; thus, providing short educational opportunities with limited disruption to their daily schedules is important (Line 98).

### Characteristics of the individual

#### Knowledge and beliefs about the innovation: give us knowledge and we will disseminate

Participants’ positive attitudes toward, and value placed on, this innovation was commonly discussed. Specifically, many found it important to educate patients about TB, differentiate between TB infection and disease, and explain why TPT is crucial even when patients are not demonstrably sick (Line 160). Healthcare workers were keen to learn more about TPT and if given sufficient training and resources agreed that a systematic implementation approach is needed for success (Line 130, 133,135, 139, 142, 146).

#### Self-efficacy: in order not to make a mistake, let us not do anything

Self-efficacy, or an individual’s beliefs in their own capabilities to execute courses of action to achieve implementation goals, [11] varied between different types of HCWs. Some interviews centered around providers—mostly nurses—not wanting to “do anything wrong” and, therefore, defaulted to doing very little regarding TPT (Line 272, 274, 275).

Disempowerment and lack of clear TB and TPT policies have often led to suboptimal implementation (Line 277). Despite the lack of self-efficacy discussed, many participants stated nurses and clinics are better suited to lead TPT program implementation because nurses spend more time with patients than doctors, nurses are knowledgeable on current TPT regimens, and they are often more adherent to guidelines than other clinical cadres. Structurally, clinics (staffed by nurses) are also more accessible to patients (Line 281, 280, 279). Currently, nurses seem to be doing the bulk of work around TPT, so providing nurses leadership roles and resources necessary to lead TPT programs could improve its effectiveness (Line 284, 281).

### Process

#### Champions: necessary in the clinic and the community

Having champions—both HCW champions as well as community champions (i.e., grandmothers, elders, chiefs)—was discussed as an important puzzle piece to support TPT implementation. One individual said: “nurses are far better at screening than doctors and I know the way our system works here, an intentional TB screen is supposed to happen for every patient. And I think it does happen actually quite well because it's part of the triage process [in the outpatient department at the hospital].” (Doctor, Male, Line 83).

The description of nurses being well trained to follow and implement guidelines—when given the resources to do so, being respected and knowledgeable (Line 191), and having a program that is integrated into a triage process—helps ensure that it gets completed without adding too much additional strain to individuals or the organization. In addition to nurses as being potential champions to successfully launch a TPT program, elders were also thought to be potential champions.

## Discussion

There are multifaceted barriers to overcome prior to implementation of an effective TPT program in this setting such as optimal HCW knowledge around TPT, intervention design and packaging, and increased resources. However, leveraging facilitators such as positive HCW attitudes, existing partnerships and champions, and dedicated TB nursing staff may improve implementation and program sustainability. Improving HCW knowledge regarding efficacy of TPT is necessary prior to effective implementation of a TPT program, especially as new regimens such as shorter 3-month combinations of isoniazid and rifapentine (3HP) are becoming more widely available and recommended. An identified enabler was that HCWs have positive attitudes towards learning about TPT effectiveness and how to rule out TB. Providing systematic support through simple and clinically relevant documentation workflows could also enable successful program implementation. Therefore, providing education with high engagement, that is free to access repeatedly, and available at the point-of-care is ideal [14, 15]. Finally, ensuring the TB program is nurse-led and clinic-based is important for feasibility, cost, and scalability [16, 17]. In summary, participants were enthusiastic but felt the need for more knowledge, clear guidelines, specific workflows, and continuous momentum. Table 4 describes next steps for program implementation.

**Table 4:** Next steps

Steps to take	Time frame	Construct
Present the importance of data-driven work that can support clinical care and not just be extra paperwork	2–3 months	Design packaging
Ensure senior management are on-board with the program and up-to-date with recent guidelines	2–3 months	Leadership/relative priority
Hold monthly, rotating journal clubs for doctors and nurses to discuss case studies and evidence for TPT and guidelines	2–3 months	Access to knowledge
Hold community events and meetings with community stakeholders to increase awareness about TPT and decrease stigma associated with TB and HIV (perhaps males)	2–3 months	Culture
Ensure pharmacists and clinics can adequately stock TPT medications	2–3 months	Implementation readiness
Ensure regular communication through WhatsApp groups and email lists from pharmacists to clinic teams about TPT stock supply in the district	2–3 months	Implementation readiness
Provide simple point-of-care guidelines and visual algorithms in clinics and use during trainings	2–3 months	Knowledge/evidence
Using local champions and community leaders to recruit community members, host regular community sessions on importance of TPT and why asymptomatic people should screen for TB and initiate TPT	3–6 months	Knowledge
Engage HCWs in small group trainings (or 1:1) about the evidence of TPT	3–6 months	Evidence, knowledge
Hold regular in-person/virtual trainings for nurses on TB infection, diagnosis, and TPT dosing to increase confidence in ruling out active disease and initiating the TPT dosing	3–6 months	Self-efficacy/knowledge
Improve engagement with CHWs for TB programming	3–6 months	Culture/implementation readiness
Take advantage of existing nurse champions to lead TB programming	3–6 months	Champions/implementation readiness
Identify at least one community medication pick up point for each clinic to reduce travel distance for patients to collect medications	3–6 months	Design quality/packaging
Identify one community champion per clinic and train them on mobilizing the community around the importance of screening and testing for TB, de-stigmatizing TB and HIV, and organizing TPT awareness campaigns	3–6 months	Champions
Improve consistency of internet connectivity at clinics for trainings, access to information on TPT, and sharing of data	6 months–1 year	Implementation readiness
Engage policy stakeholders at the regional, provincial, and national level on integrating TPT into clinical practice guidelines, educational preparation for clinicians, and continuous professional development activities	6 months–1 year	Champions

Central to any intervention is its design and packaging. Similar to other studies assessing TPT in the Eastern Cape [18–20], participants in this study reported lack of standard documentation for TPT to be one key barrier to implementation and critical to program design. Designing thoughtful and simple documentation systems is essential since often “what gets measured gets done” [21]. Ensuring the program is patient-centered and aligns with the WHO’s End TB Strategy Pillar One, which “puts patients at the heart of service delivery,” is also important [22]. Guaranteeing patient voices are heard and involved in the design of the program and provided options for TPT (when possible) is an important next step in this context.

Additionally, the availability and need for resources and competing priorities were common barriers discussed. Research in TB and primary care shows that nurses and non-physician clinicians are excellent at following clinical guidelines and they provide quality care in resource constrained settings [14, 23–26]. Additionally, care cascades for TB (similar to HIV 90–90-90 targets) have been difficult to utilize in this setting for numerous reasons such as lack of documentation for TPT, or unclear eligibility requirements, and thus inability to determine if a person should or should not be included in a “step” of a cascade, which participants discussed as a potential barrier [18–20]. However, cascade indicators are starting to be used in TB care more, though less in preventive therapy, potentially due to lack of quality reporting mechanisms [8, 27–29]. Ultimately, shifting the priority from a treatment-focused approach to prevention—in nursing and medical education, and in point-of-care algorithms and documentation systems—is necessary to transform comprehensive TB care. This is especially critical as South Africa has recently released updated guidelines greatly expanding TPT eligibility [30].

## Limitations

Interviews were conducted with a limited range of HCWs and limited scope of differing experiences. It would be prudent to further these data with community member and past or current patients with TB input. Finally, reliance on a pre-determined CFIR coding scheme could have led to authors missing pertinent data.

## Conclusion

Healthcare workers need increased knowledge regarding TPT prior to implementing a comprehensive TB program, despite positive attitudes and beliefs about TPT. Furthermore, additional resources—time, trainings, and contextualized evidence—are needed to prescribe TPT more broadly. Tangible resources such as suitable documentation systems and funding to administer TPT programming are also critical to sustainability.

### Supplementary Information

**Additional file 1:** **Supplement 1.** Healthcare worker interview guide on knowledge, attitudes, and beliefs regarding TB preventive therapy.

**Additional file 2:** **Supplement 2.** Exemplar Quotes Per Consolidated Framework for Implementation Research Domains and Constructs.

## Data Availability

The datasets analyzed during the current study are available from the corresponding author on reasonable request.

## Abbreviations

TB: Tuberculosis
TPT: Tuberculosis preventive therapy
KSD: King Sabata Dalindyebo
SRQR: Standards for reporting qualitative research
CFIR: Consolidated framework for implementation research
LMICs: Low- and middle-income countries
