# Supplementary material for: Healthcare worker perceived barriers and facilitators to implementing a tuberculosis preventive therapy program in rural South Africa: a content analysis using the consolidated framework for implementation research
Source: Implement Sci Commun. 2023 Aug 30;4:107. doi: 10.1186/s43058-023-00490-8 (PMC10468851; doi:10.1186/s43058-023-00490-8)
Supplement: Supplementary file 1 — Additional file 1: Supplement 1. Healthcare worker interview guide on knowledge, attitudes, and beliefs regarding TB preventive therapy. [file 43058_2023_490_MOESM1_ESM.docx]

Supplement 1. Healthcare worker interview guide on knowledge, attitudes, and beliefs regarding TB preventive therapy

1. **Introduction and welcome.**

Good morning/afternoon, thank you so much for agreeing to be interviewed today. My name is [X], and I’m a researcher that studies TB at [X]. We want to know about your experience in screening and prescribing TB preventive therapy and the challenges you face as a healthcare worker in providing TB preventive therapy. [*We recently did a study about TB preventive therapy among household contacts of TB patients in the [X] sub-district. We found that few household contacts were screened for TB disease (39%), and that less than 1% were prescribed TB preventive therapy.]* We will be recording this interview, so that later on, we can transcribe (write down) your words to learn about different people’s perspectives and bring them all together to try to understand different people’s points of views about TB preventive therapy. I also might take some notes while we speak in case I want to ask more about your answers. Is it ok for us to record and take notes today? [Give time for participant to answer]. If you ever feel uncomfortable, we can stop the interview at any point. Thank you.

1. **Introductory questions.**
2. Can you tell me about your professional background?
3. How long have you been involved with TB activities in [x], or in other areas?
4. Can you tell me about your views on whether or not TB preventive therapy is an important part of TB care?
5. What is your knowledge about TB preventive therapy preventing TB from spreading or worsening in individuals infected with TB?
6. What kind of education have you received about TB preventive therapy during school or any additional courses you have attended?
7. **Main, topic-focused questions. (Knowledge, attitudes, beliefs)**
8. Can you describe reasons why health care providers might or might not screen people for TB?
9. What do you think makes screening household contacts difficult?
   1. Can you tell me more about that?
10. What do you think makes screening household contacts easier?
    1. Can you tell me more about that?
11. What has your experience been in screening individuals for TB preventive therapy?
    1. Are there certain groups that are easier to screen (i.e. pregnant women, children <5, people living with HIV, household contacts)? Why?
    2. Are there any groups that you find more difficult to screen? Why?
12. *What has your experience been in prescribing or providing TB preventive therapy to individuals? (perhaps people living with HIV, household contacts of TB patients (or DR-TB patients), pregnant women, children, etc)*
    1. Specifically, can you walk me through what a visit is like when you diagnose a patient with TB?
    2. How do you elicit information about their household contacts?
    3. What do you find is most challenging in screening household contacts? [availability, children, finding out HIV status of contacts, etc]
13. What do you think are the main challenges for **health care** **providers** to prescribe TB preventive therapy?
    1. Do you have any examples you can tell me about?
14. If we/you want to provide TB preventive therapy to all eligible individuals in the [x] sub-district, what would you recommend we/you do?
    1. Could you give a few concrete examples of what would help/facilitate TB preventive therapy prescribing for **health care providers**?
15. Based on your interactions with patients, what challenges do you see them experiencing with TB preventive therapy?
    1. What could we/you do – as **health care providers** – to improve the experience for patients we/you want to initiate on TB preventive therapy?
16. **Follow-up questions.**
17. Would you be interested in learning more about TB preventive therapy prescribing, and why?
18. Are there any specific topics about TB preventive therapy specifically that you would like the most information on?
19. Could you recommend a good way or best format to teach this information to health care providers?
20. **Closing questions.**
21. Is there anything else you’d like to tell me today that I haven’t asked about yet?
22. Do you have any questions about the interview?
23. **Closing remarks.**
24. Thank you so much for participating today. This information is helpful for us understand more about healthcare providers perspectives about TB preventive therapy. We will be transcribing and analyzing the interviews we have conducted, and we will share the information back with you soon.
