# Supplementary material for: Healthcare worker perceived barriers and facilitators to implementing a tuberculosis preventive therapy program in rural South Africa: a content analysis using the consolidated framework for implementation research
Source: Implement Sci Commun. 2023 Aug 30;4:107. doi: 10.1186/s43058-023-00490-8 (PMC10468851; doi:10.1186/s43058-023-00490-8)
Supplement: Supplementary file 2 — Additional file 2: Supplement 2. Exemplar Quotes Per Consolidated Framework for Implementation Research Domains and Constructs. [file 43058_2023_490_MOESM2_ESM.docx]

Supplement 2. Exemplar Quotes Per Consolidated Framework for Implementation Research Domains and Constructs

| **Intervention Characteristics** | | | |
| --- | --- | --- | --- |
| Design quality and packaging | | | |
| Participant demographics, quote line number | Direct quotation | | |
| Data Capturer: Male, 20s; 2 years as data capturer at this hospital/catchment area; Line 38 | People are doing good, but it's a matter of record keeping and, and, the quality of that. | | |
| Hospital MO: Female, 30s; MO for 15 years; Line 49 | I think change management also takes forever because if you sort of say, "Listen TPT is important. Tomorrow we do it 100% right". It's not gonna work like that. It's gonna take a while because you first need to start talking about it. And then the registrar [junior doctor] in the meeting’s like "Oh yeah, I forgot about TPT". And then they go to [one] Clinic and there's an extra hour, they don't know what to do. "Oh, TPT guys. Did somebody talk to you about it?" and then we start talking about it. And then we... The more we talk about it, the more we get aware of it and we sort of read the guidelines a bit better. We start thinking "How can we implement that?" and then... So I think it - I mean I have a lot of grace that it's gonna be a process, that it's gonna - we start... I think the first step is just get people to believe in it and start talking about it. Keep on talking about it… | | |
| Hospital MO: Male, 30s; <5 years at this hospital; Line 52 | And so TB nurses should be able to spend more time with each patient that comes through their, through their room. And in fact, it might be as simple as you know - well not the checklist exists, I guess. It’s on the back of the, the TB registration isn’t it. | | |
| Hospital MO: Male, 30s; <5 years at this hospital; Line 54 | An algorithm, a simple kind step by step algorithm needs to be drawn up with as few options as possible. Obviously keeping it safe uhm that, that can be as sort of - with as little jargon and as little specialized training required as possible. | | |
| Hospital MO: Male, 30s; <5 years at this hospital; Line 55 | I think that keeping it as simple as possible...I think our clinics don’t have access to online resources uhm and I think that you need, I suppose you need buy in from the clinics. That’s where most patients have access. Ideally you want to be able to get to the patient as opposed to the patient coming to us, but that has ethical and logistical challenges obviously. Uhm, and I think having the - at each facility almost, you know start small. Start at one, one level, one site. Trial it here where we can you know - three months in, six months in - try and see what challenges and you know redesign the system to a…cycle essentially isn’t it? It’s a quality improvement cycle where you need to keep cycling to, to simplify and make it more, as effective as possible. Uhm then at each facility to, to champion one, one clinician, one nurse or one doctor who's going to not do it all, but is going to push it [the intervention] at the facility. And it doesn’t necessarily have to be a clinical manager or an operational manager. It can be any you know, any clinician of any level who’s got a passion for TB treatment. | | |
| Hospital MO: Female, 30s; MO for 15 years; Line 50 | they [Patients] don't come back…people don’t feel well and they get sick they will come in. So I think for me it's about "Here's a child, less than 5, no active TB". [Give them] six months treatment. There you go. | | |
| Pharmacist: Female, 30s; at this hospital for 3 years; Line 60 | pre-packing would be a good system to decrease congestion at the hospital because if you don't need to be here then don't be here, you know. So, if it's just for TPT, but the clinics for whatever reason cannot keep the medication or procure it - then at our level we would have to pack it the way we pack the ARV's and send it off to the clinics and be like "Okay, this is patient X's medication. Patient Y which..." you know with instructions and everything. | | |
| Hospital MO: Male, 30s; at this hospital for 7 years; Line 63 | I think it has to be a program that starts and is based at the clinics. And that we come in and we are support for them. We field questions, we help them with difficult cases, we sort of provide more of a consultation basis, rather than a, we're coming in and we're saying “This is our program”. So, I think it's important for us to, to do that, uhm so that they also feel as if they've got better capacity, they feel strengthened, they feel uhm yeah, they feel more equipped to actually uhm deal with those kinds of things. | | |
| Complexity | | | |
| Hospital MO: Male, 30s; at this hospital for 7 years; Line 13 | “It’s one thing getting the information, it’s another thing actually tracing uhm which I think that’s where the difficulty is. And I don’t think that that’s a personal issue in terms of the issues that they’re not doing the work, it’s a matter of - like I said - access. How do you actually then go about finding those contacts and what do you then do?” | | |
| Hospital MO: Male, 30s; at this hospital for 7 years; Line 26 | “So the knowledge around who’s eligible, who’s not, proper diagnostic screening, as well as I think, knowledge around which drugs are available. When we prescribe them, who gets them? What do we do when the contact actually comes? Because it’s one thing to say we’re doing [screening] the household contact, but if you don’t know actually the steps to take when the contact is in front of you, then that becomes an issue.” | | |
| Hospital MO: Male, 30s; at this hospital for 7 years; Line 16 | Definitely improving capacity at community level, primary health care level. Uhm so improving capacity uhm for the nurses, linking care as well in terms of how do I link this in with the already existing programs that are running at the clinics - whether its family planning, immunizations for children, antenatal visits for adults - trying to make it seem more accessible and easier for the people involved? Uhm, involving the community health care workers because it’s one thing getting everybody and being able to prescribe the therapy, but it’s one thing getting people to take it and stay on it. Uhm, I think it’s, its yeah. Uhm getting to a point of we are prescribing uhm TPT for, for all household contacts or everyone eligible is one part of it, but making sure that the people who it’s prescribed for actually stay on it, that's another issue. Uhm and I think it's a multifaceted. It has to do with different levels of care and different levels of uhm reaching the patient where they are and uhm contextualizing their care. If you know that it's one of the areas in which, you know, okay there’s females of childbearing age in this household offer them the family planning with their IPT uhm or their TPT. Uhm if you know that there’s children possibly receiving immunization - children under 6 and under - offer them their uhm TPT with their immunization program. If there is gogo with her chronic hypertension, offer her the care and link it with the care that she’s already getting at the clinic. So don't make it a concept of its own, but sort of work it into the pre-existing programs that the community’s already engaging with or engaging in rather than then making it another task of, “Oh, on top of your family planning visits you also need to go to the clinic to go get your TPT” | | |
|  | | | |
| Evidence strength and quality | | | |
| Hospital MO: Male, 40s; 15 years at this hospital; Line 70 | not dealing with a latent TB is basically the reason why we're still not improving things. So, I think that - my sense is - that unless you want to keep on playing whack-a-mole, you have to do it [TPT]. It's just a matter of doing it thoughtfully and doing it systematically. | | |
| Hospital MO: Female, 30s; MO for 15 years; Line 79 | So I think there's not a disbelief in the TPT, but is it applicable and is it practical in this setting? - was more the question. So I think out of that debate I got, you know TPT it does work. Okay. We need to start doing it, but how are we gonna implement it on a national scale? And I think about six years ago we were still struggling to get our drug sensitive people all of them on HIV because then the guidelines changes that all TB patients needs HIV treatment. So when I was having meetings with TB point it was about which patients actually was HIV positive. And they needed ARV's. So for me I think we as a team decided "Let’s just get the first things in place". Not even all our TB patients get ARV's. We're not even talking about TPT. So I think what we also need to understand about rural context is that we are so far behind. | | |
| Pharmacist: Female, 30s; at this hospital for 3 years; Line 81 | when you read about TB in this country they mostly focus on like active TB, DSTB, DRTB. You don't really get a lot of data where they talk about TB, about TPT and the success rate and all of that. So I think there also is a lack of interest because there isn't a lot of readily available information. | | |
| Hospital MO: Female, 30s; MO for 5 years. Family Medicine registrar; Line 65 | so I think when the conversation around the change in guidelines or the recommended changing guidelines around preventative therapy was, at med school and all the way through I didn’t realize that - and I think a lot of people don’t realize - that when we give preventative therapy it’s not a protective... It’s a, it is protective at the time, but it also treats the latent TB. And that totally changed my perspective. And I'm like “Oh wow. This needs to happen”. Because when I realized… I think you don’t know that anything is possible. Like when I mentioned to nurses that “Oh, did you know that like in other countries they've eliminated TB?” They're like “No they didn’t”. I'm like “No, it’s true. Its sounds crazy, but it’s true”. | | |
| Hospital MO: Male, 40s; 15 years at this hospital; Line 68 | I know regard TBT as a critical part of treatment of - and management of TB can getting control of the TB epidemic in the country. And I think that uhm, I think that in my early days - I mean certainly it wasn't part of what my training was about, it's only in the last three or four years that I’ve actually realized how important it can be. And I think part of the issue is partly because I think that, that uhm, I think it's certainly not part of what we get taught as undergraduates as- apart from in uhm, in kids who've been exposed to TB. So I think that - that... And my undergraduate degree it wasn't really something that was focused on particularly. I think partly because you know WHO policy has been said that it's not, there is no space for it in uhm, in kind of high burden countries. | | |
| Hospital MO: Female, 30s; MO for 5 years. Family Medicine registrar; Line 66 | People aren’t so good. And I think maybe people are more weary of like if you start someone with low CD4 count on HIV's, often they come back with the TB-IRIS having suddenly their TB wakes up that we could diagnose before so. And they don’t wanna be that person who causes INH resistance because they were on it for a couple of months, so yeah. I think there's enough to say like “Oh, never mind”. They don’t understand - and I, also me I don’t understand - the weight of INH. | | |
| Hospital MO: Male, 40s; 15 years at this hospital; Line 69 | I think a sense of my worry about IPT has always been that you might give it to someone who then - or gets rid of their latent TB, but then gets reinfected six months later, a year later. And so you kind of wonder if the, if the benefit of - and the cost benefit, but also the risks of side effects are as beneficial when it's not having a long lasting effect. So I think that... So and so uhm, I think that was part of why I was hesitant before. I think that - I do think... I understand the rationale from some people who worry, but that giving IPT is basically gonna just be throwing stuff down the drain because people will be reinfected again. But I think, I think that if it's done as part of a - of a comprehensive program and a thoughtful program, I think it can be very beneficial. | | |
| Hospital MO: Male, 40s; 15 years at this hospital; Line 71 | we've had a fair debate in the team uhm, about IPT. And I think that there's quite strong opinions within the team and in - within South Africa - about whether IPT is beneficial or not. And I think, I mean... So and because there's been this kind of debate and arguing about it, we've kind of been finding ourself sitting on the fence. | | |
| Hospital MO: Male, 40s; 15 years at this hospital; Line 74 | I think that there's certainly education that can be done and because it's not been implemented very clearly and effectively and because I think the policy hasn't always been clear I think that policy uncertainty has meant that there has been uncertainty amongst clinicians and there hasn't been a like a clear approach. And so people haven't really implemented in a good way yet. | | |
| Hospital MO: Male, 40s; 15 years at this hospital; Line 68 | I know regard TBT as a critical part of treatment of - and management of TB can getting control of the TB epidemic in the country. And I think that uhm, I think that in my early days - I mean certainly it wasn't part of what my training was about, it's only in the last three or four years that I’ve actually realized how important it can be. And I think part of the issue is partly because I think that, that uhm, I think it's certainly not part of what we get taught as undergraduates as- apart from in uhm, in kids who've been exposed to TB. So I think that - that... And my undergraduate degree it wasn't really something that was focused on particularly. I think partly because you know WHO policy has been said that it's not, there is no space for it in uhm, in kind of high burden countries. | | |
| Hospital MO: Female, 30s; MO for 15 years; Line 78 | so I think, I think it's difficult - you know because I have such a history uhm, to say it in one sentence - but basically I think when I started uhm doing TB and Drug Resistant TB there was still a lot of research going on. And I think our role models in the country - our top researchers was talking about "Is it worth it? Is it not?" and the research started coming out and there was a lot of studies. "Is it happening? Is it not happening? Is it worth? Is it not?" So there was a lot of uhm sort of different opinions on that I think. That's where I started. So I think I was already biased not to really... I mean when you hear your researchers and guidelines say that you should do this, you believe in it. So I think there's a background in South Africa of you know is it working? Is it not working? And sometimes you struggle to get rid of that stigma. So the first time that I was a little bit convinced that it perhaps works was uhm when I was in a HIV TB conference and that was in - I think around 2014 or 15. And in that conference there was actually uhm a debate between two specialists. The one for TPT and the one - tother one against TPT. Uhm and it's two of our top researchers in the country. I can't remember who it was, but uhm the two gurus. | | |
| Hospital MO: Male, 40s; 15 years at this hospital; Line 70 | not dealing with a latent TB is basically the reason why we're still not improving things. So, I think that - my sense is that unless you want to keep on playing whack-a-mole..., you have to do it. It's just a matter of doing it thoughtfully and doing it systematically. | | |
| Outer setting | | | |
| Needs and resources of those served by the organization | | | |
| Hospital MO: Male, 30s; at this hospital for 7 years; Line 166 | | uhm, really uhm I’m very worried about our people - knowing their socio-economic levels, knowing about their education - low educational levels. It’s, they are the very people really they are being infected by TB. And unfortunately, they are the people with no - I mean they are always overcrowded, because they are always stay with grand and grand grand children in one place. It's so pathetic - honestly - about this group. Because they really need somebody who will be…oh I don't know how to put it. Who will be patient. 00:41:16-0  Interviewer: Yes of course. 00:41:16-0  Interviewee: Who will be patient and have a heart, I mean to understand that… you know I always tell even the staff members “You know when somebody comes through the door, think of someone who doesn’t know… think of somebody who just comes into darkness, doesn't know where is the entrance, where is”. The first time the patient comes to the clinic, doesn't know where to go. You have… even you can't say you can't talk. You talk from the entrance of the patient until the exit of the patient. When the patient comes through the door you tell her - the patient will say “Can we sit there?” You direct because that person doesn't know the place. You tell the patient “I’m so and so. Uhm what are you coming for? Are you coming for registration? You sit here. Then when you sit here you do this, you go to there, from there you go there” - directions. 00:42:23-5  Interviewer: yes, yes. And give them warm invitation, yeah. Yes welcome them. Yeah, yeah, not just rush, rush, rush and take your time. 00:42:32-5  Interviewee: And then that… and then when they come in then you’ll… the patient is relaxed. | |
| Hospital MO: Male, 30s; at this hospital for 7 years; Line 165 | | They need to be told that on taking, why they take this treat… what is this treatment name, why they take this treatment, what will be expected from this treatment and the outcome of it | |
| Clinic nurses: Females, 30s;  **Two interviewees; Line 167 | | Challenges is more for finding TB contact because long distance, no transport. | |
| Clinic nurses: Females, 30s;  **Two interviewees; Line 168 | | So due to financial problems some of them won't have the chance - they don't have even the money for the transport. | |
| Clinic OM: Female, 40s; Professional nurse; 6 years at this clinic; Line 169 | | It's for them to come in. It was easier when - it's the transport also that is also the challenge because as much as we have community health care workers, there are persons they can't reach. | |
| Hospital MO: Male, 30s; <5 years at this hospital; Line 174 | | Access. I think that we don't have access to contacts very readily uhm because we don't go into the communities. And I think that in a system where the ward based outreach teams are functional or where the community health worker systems are functional - and they probably are in some of our communities - those are well placed to, to provide screening. And in fact if we had a strong ward based outreach team that might be an ideal way to, to connect to - you know each of our newly diagnosed TB patients to a screening program of some sort. But they’re so overwhelmed by everything else. There’s so much that needs to happen uhm along with in rural areas the, the distances between houses in so vast and, like I said, you know access in terms of patients getting to us and us getting to patients to, you know to expect someone to walk tens of kilometers every day and still be effective is - or efficient - is, is… | |
| Clinic OM: Female, 40s; Professional nurse for 25 years; OM for 1 year; Line 176 | | So contacts are hard to screen uh because they're hard to reach. 00:07:54-6  Interviewee: yes. 00:07:55-3  Interviewer: what else? What else makes it hard to find the contacts and to screen them? Is that the main reason? 00:08:02-8  Interviewee: uh it's because of this area it's too scattered. 00:08:06-1  Interviewer: mmm. 00:08:07-5  Interviewee: each village is far away from each other. 00:08:11-2  Interviewer: yeah. 00:08:12-2  Interviewee: and because of there is this uh problem with the roads is also another issue. But what I usually do, if I come in contact with a patient that I screen and I take the sputum and I see that the patient is positive, I used to give the bottles. And what I get I only get that "No, they said they don't have the sputum’s. They can't cough for me". 00:08:42-2  Interviewer: yeah, yeah. 00:08:43-7  Interviewee: I tell the patients "No, tell them to come here to the clinic". "But I don't have money to let these people to come". "How many of them?" - "Its plus, minus five people in the house". | |
| Clinic OM: Female, 40s; Professional nurse for 25 years; OM for 1 year; Line 177 | | I will just specify that I think it's only the age group that will just give the problem. 00:15:58-3  Interviewer: okay. 00:15:59-1  Interviewee: especially the teenagers. 00:16:01-3  Interviewer: they would give a problem? 00:16:02-4  Interviewee: they will give the problem. With the elder people you will never get it. 00:16:05-4  Interviewer: they'll take, they'll take the medicine all day? 00:16:06-9  Interviewee: they will take it all. 00:16:08-5  Interviewer: okay. So why won't the younger people take it? 00:16:10-7  Interviewee: because they will tell you that "I'm not sick. Why must I take it?" 00:16:14-5  Interviewer: yeah. Okay. Alright. Okay. 00:16:17-8  Interviewee: they will wait until they get sick. | |
| Hospital MO: Female, 30s; MO for 5 years. Family Medicine registrar; Line 181 | | You can always tell the TB contacts in OPD because it’s a mother with about five children who all look totally, totally well. So yeah, kids I think are easy. Uhm, men. Getting men. | |
| Data Capturer: Male, 20s; 2 years as data capturer at this hospital/catchment area; Line 183 | | Yeah, it's not filled in. But if for instance you go to pharmacy, you'd find that there is a record of that patient getting IPT... 00:05:18-9  Interviewer: Of them getting the IPT. 00:05:18-6  Interviewee: So first of all we just have to be recording of that. And which is, I don’t know if there's any source document - whether be at pharmacy, whether be at the ward - that kind of says that if a patient is given IPT at the ward by the doctor or - I don't know how the systems work of, of, of the pharmaceutical department - but kind of have a source document where they actually record having given the patient. | |
| Pharmacist: Female, 30s; at this hospital for 3 years: Line 186 | | so the clinics - unlike us, we have the option of ordering from the depot or ordering from the supplier through the depot. The clinics don't have that option. They have to order from the depot. 00:30:42-3  Interviewer: okay. 00:30:41-4  Interviewee: so you'll find sometimes we have certain medication, the clinic doesn't have it. Because we've ordered from the supplier, but through the depot. So we can put direct pressure on the supplier to issue the stock. 00:30:53-4  Interviewer: I see. 00:30:54-3  Interviewee: the clinics cannot do that. They have to rely on the depot putting pressure on the supplier to get the depot the stock and then you know they roll - they can uhm receive the stock. 00:31:03-7  Interviewer: yeah, yeah. 00:31:05-2  Interviewee: so... But I mean at the clinics they do have a subdistrict pharmacist, but I really can't say much because I - beyond bailing out the clinics here and there, I'm not really sure what their stance situation most of the time is. | |
| Inner Setting | | | |
| Readiness for implementation | | | |
| Hospital MO: Male, 30s; at this hospital for 7 years; Line 212 | | Unfortunately, we don’t have 24-hour lab services. So, if you see a patient after hours and you’re concerned about TB, unfortunately they'll have to sleep over in that space with other patients…which is quite a risk, but we can’t send them home because there's no transport and it’s quite far out for most of our patients. So, until we get the GeneXpert results then they're sleeping over in OPD. | |
| Data Capturer: Male, 20s; 2 years as data capturer at this hospital/catchment area; Line 215 | | So basically I’ve never came across a source document that has been produced by DOH saying that this is specifically for IPT. Because I think the last time we had a meeting, it was kind of like uhm all these different sections, if they happen to do - issue IPT - they'd have to kind of create like a textbook or notebook for themselves where they kind of like record keeping it because the way it's supposed to be done whereby we get the records of the IPT from the patient folder, is not happening. | |
| Clinic OM; Female, 40s; Professional nurse >10 years, OM for 1 year; Line 219 | | For some reason people with uhm pregnant people, because there is a guide for you to fill in that you have done that, you understand what I mean?  Interviewer: I do.  Interviewee: So in that case you can't omit it. For instance if you look at uhm our patient’s file now -, the new patient file we have, single file - it has got that part of TB screening. So you have to tick. You understand, so you can't miss it | |
| Hospital MO: Male, 30s; at this hospital for 7 years; Line 214 | | making access easier. I think bringing the drugs closer to home, maybe bringing the drugs to their homes. Uhm I can't, I can't imagine for, for myself it's easy, because I, I know the implications, I understand the implications. I don't mind travelling because I also have the means to do so uhm for a vaccine or for uhm something that's preventative. I don't mind doing that, but then asking somebody to travel 10km, 15km uhm every four weeks or every three weeks to the clinic to get meds that they don't understand they need, or they don't feel they need, or they don’t perhaps appreciate just how much they need - which is also like I said, not at all their fault. I completely empathize and understand why - uhm I think we need to make it a point to try and improve that access. Uhm to try and say “Okay, maybe we'll set up hubs that are closer to the homes. Maybe we'll make it, the drugs need to be accessible at the clinics. Uhm, maybe we'll work on a courier system of the health care workers actually delivering the meds to the homes. Uhm let’s try and make it easier for you to actually get the meds” uhm so that it's not an added task on the already growing task of your everyday life, uhm I need to do this and that and that and that and now I need to go collect treatment for an illness I don't have. | |
| Hospital MO: Male, 30s; at this hospital for 7 years; Line 247 | | Access. Uhm I think gaining access to the household as it is for health care providers uhm and the contacts gaining access to uhm health care or to the healthcare facilities is a big thing. If you've taken your granny to the hospital and the trip has cost you R600 uhm to get them to the hospital and then they get diagnosed with TB, and you've got eight other people at home - who are well otherwise or perceive themselves to be well otherwise - uhm the investment in trying to get those eight other people to the facility for screening or trying to get someone from the facility to that home to screen the household sort of kind of does not seem to be in keeping with uhm the matter or the issue at hand. I think for a lot of people it's a matter of its taken so much to actually get to the healthcare facility, the idea of now bringing well people to come get screened doesn't quite make sense. Uhm and also for health care workers who like I said were stretched quite thin, uhm getting people out there uhm is a bit of an issue. Uhm, community health care workers or community care workers are, I think a large part of the solution or a helping hand in doing this. But even they have been stretched quite, quite thin. Uhm, we don't have nearly enough uhm - especially with remote areas like we live in here uhm - trying to get to the last house on the hill may take the whole day. Uhm and how many households do you then reach on foot uhm across the valleys, over the hill, crossing the rivers? It's, it's, it seems like a bit of a Jumanji game uhm, so yea. | |
| Hospital MO: Male, 30s; 5 years at this hospital; Line 232 | | and the other thing which is uh already in our health system, but again arguably is not very well managed or capitalized on would be the community health workers which are - their job description is exactly that. To go out into the community and promote health in whichever form is dictated to at the moment. But in general, uhm majority of them are more based in the clinic, which is not the point. But I mean that, that could be an access point. | |
| Clinic OM: Female, 40s; Professional nurse for 25 years; OM for 1 year; Line 240 | | I think if we can get uh more staff including the vehicle or a bakkie just to go and reach them. 00:12:03-8  Interviewer: to reach them yeah. 00:12:04-9  Interviewee: it's much better. 00:12:06-9  Interviewer: yes, yes. 00:12:07-2  Interviewee: you get more staff and not just more staff, skilled staff. 00:12:12-2  Interviewer: yes. And skilled in what way? 00:12:14-0  Interviewee: in professional nurses. 00:12:16-2  Interviewer: okay. Professional nurses that are trained in preventive therapy. 00:12:21-2  Interviewee: yes. Not just uh community health care workers. | |
| Pharmacist: Female, 30s; at this hospital for 3 years; Line 242 | | I think it would be easier if we had more community health workers because they live in the community. So they know who is who and who lives where, even if they move around. They know person A is actually from that house - you know on top of the hill - and not this one in the valley. So they - community health workers have knowledge uhm - information that we don't have because we don't interact with the community as much as they do. So I think if we had community health workers I think the screening would probably be more successful because they would, they would know what questions to ask, they would literally know you know people's names and be like "I'm looking for the family of John Michael" you know what I mean. | |
| Pharmacist: Female, 30s; at this hospital for 3 years; Line 243 | | I know that they do go around you know and - I'm not sure if the ones here actually do screening - but I know they do go around and help us you know patients that lost a follow up, but I think just as we need training in TPT and why we do it, I think the same would have to apply to them so that they can understand why they're actually going house to house and you know having conversations with people. So I think that's the first thing. But also they would need to be equipped in terms of resources. So you know, like I said most of the houses are a bit of a distance apart, so we'd need to equip them with vehicles, uhm with like actual you know stationary because as I’m sure you've noticed this is like a really impoverished community. So you can’t tell someone "Oh I need you to go screen person A, B and C" and you don't even get them like just basic stationary. | |
| Pharmacist: Female, 30s; at this hospital for 3 years; Line 246 | | I think in a situation like this pre-packing would be a good system to decrease congestion at the hospital because if you don't need to be here then don't be here, you know. So if it's just for TPT, but the clinics for whatever reason cannot keep the medication or procure it - then at our level we would have to pack it the way we pack the ARV's and send it off to the clinics and be like "Okay, this is patient X's medication. Patient Y which..." you know with instructions and everything. Obviously that increases the burden on the clinics because now they probably have to have someone dedicated to just keep giving out packs, but uhm I think maybe that's where you know they probably use the ENA's or the EN's uhm you know and also in giving out the pack if you've educated the ENA's and the EN's they can also you know just do a quick education thing with the patients and be like "Oh, this is your medication. It's you know, it's uhm for TPT. This is how it works. This is what you need to do. If you have any issues just you know come back to us. You don't have to go to a hospital" | |
| Clinic OM; Female, 40s; Professional nurse >10 years, OM for 1 year; Line 250 | | The thing is, with the community health workers there's not much knowledge. I mean their training is limited. So when it comes to assessment and clinical assessments of patients, they may miss some of the things. The work you can do to give them. Like for instance we are, they are helping us with Vitamin A because they just look at the pat - at the children's cards and see that this person must not be given… but in cases where there will be a clinical assessment, it's not gonna be easy for community health workers to do that, to assess - I mean to assess those people that they are, what's their problem. | |
| Hospital MO: Female, 30s; MO for 5 years. Family Medicine registrar; Line 262 | | they are rewarded by things improving. They've seen contacts are getting screened properly so it’s like this whole feedback cycle of, of I’m not just sending my community health care workers out into the valleys for no good reason to bring kids back and I’ll be like “Oh sorry, I don’t know. I'd rather not give them INH in case they have TB and then they get resistant. Let’s just wait till they get sick”. | |
| Hospital MO: Female, 30s; MO for 5 years. Family Medicine registrar; Line 263 | | “People can do wonders with the right support. And I think a lot of our community health care workers - I mean ideally there’s a ward-based outreach team supported by the nurses, who's supported by the family… like there's - on paper - this beautiful idea. In practice it’s all a bit higgledy-piggledy and no one knows who must they report to and that was [name] whole study is “If you supervise them, do they do more? Oh yeah, they did way more. They're like changing lives”. And they don’t even realize it. That’s the coolest part.” | |
| Clinic OM; Female, 40s; Professional nurse >10 years, OM for 1 year; Line 162 | | it depends on the attitude of the person who’s seeing the patient. Because if you have got a welcoming attitude the patient will tell you everything. But if you are that person that have got that uh - what do we call it? 00:23:33-8  Interviewer: like judgments. 00:23:35-2  Interviewee: Judgementalist yeah attitude, immediately the patient comes to the door, she will she that you are judgmental. 00:23:43-8  Interviewer: Yeah of cause. 00:23:44-9  Interviewee: He knows that - or she knows that - he has got TB and he or she still think it’s a stigma. So now you look at that person. Talking to that person is very important to realize that that is something that can be cured. It's not something that will stay for long. | |
| Implementation Climate – relative priority | | | |
| Hospital MO: Male, 30s; MO at this hospital 7 years; Line 98 | | | time constraints. Uhm, unfortunately in state we have less capacity than what we ideally should have, uhm which ties into the agendas for the day uhm to try and get the primary problem sorted out. And I think oftentimes the care that we're trying to provide is not bare minimum, but certainly I will try fix what you came for. Uhm, so yeah screening programs sometimes take a bit of a back burner in, in those instances. |
| Hospital MO: Female, 30s; MO for 15 years; Line 106 | | | TPT was always on the background. Yes we know how TPT... 00:09:11-2  Interviewer: yeah, it's been on the back burner... 00:09:13-5  Interviewee: but it's not the main priority. 00:09:15-7  Interviewer: right, yeah. 00:09:16-3  Interviewee: so then basically the TLD happened. How do we switch everybody over to TLD? Okay. And I think COVID happened. 00:09:26-1  Interviewer: yes. And it's continued to be - take the back seat, yeah. 00:09:28-7  Interviewee: so basically it's a six years process and I think we had a bit of glimmer of hope - last year before COVID - because South Africa started talking about TPT guidelines and how to implement it and whatever. |
| Hospital MO: Female, 30s; MO for 15 years; Line 108 | | | post COVID crisis of having not enough doctors we're not even getting that blue TB card properly filled. And somehow the TB contacts - like I say - just always is the backburner. It's not the priority. |
| Hospital MO: Male, 30s; MO at this hospital 7 years; Line 99 | | | I think if it's there and it's in your radar and its sort of in your face, because there’s posters above quit TB everywhere, there's constant reminders and it makes it a bit easier. But once the posters come off the walls we go back to “Okay, what are you here for? How can I help you?” Uhm currently our healthcare system is in survival mode. We’re not thriving yet. So I think that's a big part of why screening programs uhm often times don't do as well as proposed. Uhm because we are primarily in survival mode trying to deal with the primary issue. Uhm, yeah and I think the lack of education around it and the comprehension of how does this actually tie into the patient who comes in as sick with TB. |
| Hospital MO: Female, 30s; MO for 5 years. Family Medicine registrar; Line 100 | | | It’s of like, because kind of the… where our conversations with most of the clinics when we go to do registrar visits, it’s kind of uhm dependent on what the flavor of the week is here at Zithulele [chuckles]. Like this particular program to - this quit TB prevention - like has its moments of like spikes. So we're like “Let’s go and educate all the clinics” and then like COVID suddenly sprang up and everyone’s like “Okay, let’s just get through that”. And so it hasn’t really been a sustained conversation. I think everyone’s just pulling themselves back into what it feels like normal life. |
| Hospital MO: Male, 30s; 5 years at this hospital; Line 104 | | | dedicated kind of teams. Perhaps, yeah. I think, I think uh clinics are busy with lots of things and I think to start try and trace people is perhaps low on their priority list. Because it’s a bit of a, it's that too big amount and to now try and uhm actually... So they're still active contact tracing. So that's what DR TB has done. Active TB tracing. Whether nurses go out and do a home visit and try and actually identify and try and bring them back and make actual active plans. And that doesn't happen with drug sensitive TB as far as I know. |
| Pharmacist: Female, 30s; at this hospital for 3 years; Line 112 | | | I think because we've just accepts that TB is gonna be here. It's part of our lives and... Well that's just my personal opinion. You know it's just like "Ag, it's just TB" you know. "It's always gonna be here. It doesn’t matter how aggressively we try and deal with it, it's just always gonna be here" so I think people have become like - I don’t wanna say complacent, but people have become like complacent about it. Like it's something that happens. |
| Hospital MO: Male, 40s; 15 years at this hospital; Line 114 | | | I think there's kind of perhaps a slight apathy or kind of - not... Or just sort of "How are we gonna do that?". Because - and I think that, that the challenge with that in a context where you're busy and you - like you... You just wanna give someone their medication and say "Great. Sort you out. Yeah" and you, you next... And so the kind of the, the amount of logistical headspace it takes, I think is a little bit too much for the average, busy, junior doctor in the hospital. |
| Hospital MO: Male, 30s; <5 years at this hospital; Line 83 | | | I think, again I think nurses are far better screening than doctors and I know the way our system works here an intentional TB screen is supposed to happen for every patient. And I think it does happen actually quite well because it's part of the triage process. |
| Hospital MO: Male, 30s; <5 years at this hospital; Line 234 | | | And so TB nurses should be able to spend more time with each patient that comes through their, through their room. |
| Clinic OM: Female, 40s; Professional nurse for 25 years; OM for 1 year; Line 238 | | | Here we rarely screen. They even sent us one enrolled nursing assistant to come and just focus on TB. |
| Readiness for implementation – access to knowledge and information | | | |
| Hospital MO: Male, 40s; 15 years at this hospital; Line 199 | | not part of what we get taught as undergraduates | |
| Hospital MO: Male, 30s; MO at this hospital 7 years; Line 198 | | But we need to get the training first really. For instance, I can't say “I’ll teach the team” for something that I don't even know. I’ve got no knowledge. | |
| Hospital MO: Male, 40s; 15 years at this hospital; Line 200 | | So I mean I think some of it's about knowledge and uhm understanding, some of it is about uncertainty around the diagnosis. I think that was probably what you alluded to in the earlier question about how easy it is - is it to make a diagnosis. And I think, and I think that actually the thing and the kids thing is probably something you know we could actually benefit from having a lecture, somebody like [name] or somebody from Cape Town doing a good thorough approach to how do you diagnose TB in kids? And I think - I mean it's, that would be a good start for our whole team to get a little bit of confidence about us. I think that the interpretation of children's X-rays is hard. And so I think that there might, I think that people are nervous about making a call in fear of making a mistake. So that they feel like "Is this TB or is this just latent TB...?" - I mean T... "Is this, can I give them TBT or am I nervous?" And I think I mean it takes experience and I think people are a bit nervous to make that call. Uhm someone like [name] who does a lot will be very capable and confident of doing, but I think that... I think that perhaps we rely too much on people with pediatric interest to make those decisions and we should be broader skills that are in our team. | |
| Implementation climate – learning climate | | | |
| Hospital MO: Male, 30s; MO at this hospital 7 years; Line 89 | | It's got to do with continuous learning, guidelines are often changing, uhm new drugs being uhm rolled out, new guideline is being rolled out, uhm growing needs as well uhm. The healthcare system is quite dynamic uhm so yeah opportunities to keep updated uhm to learn, uh never a bad idea. | |
| Hospital MO: Male, 30s; MO at this hospital 7 years; Line 98 | | time constraints. Uhm, unfortunately in state we have less capacity than what we ideally should have, uhm which ties into the agendas for the day uhm to try and get the primary problem sorted out. And I think oftentimes the care that we're trying to provide is not bare minimum, but certainly I will try fix what you came for. Uhm, so yeah screening programs sometimes take a bit of a back burner in, in those instances. | |
| Implementation climate - culture | | | |
| Hospital MO: Female, 30s; MO for 5 years. Family Medicine registrar; Line 181 | | You can always tell the TB contacts in OPD because it’s a mother with about five children who all look totally, totally well. So yeah, kids I think are easy. Uhm, men. Getting men…… | |
| Clinic OM: Female, 40s; Professional nurse; 6 years at this clinic; Line 33 | | The males. Males. Especially if they are drinking alcohol. They are the problems. | |
| Clinic Pharmacy Assistant, Pharmacy Assistant since 2019; Line 34 | | I don't know what's wrong with males, but they don't…the guys they don't take it seriously, yeah. | |
| Characteristics of individuals: knowledge and beliefs about the innovation | | | |
| Hospital MO: Male, 30s; MO at this hospital 7 years; Line 160 | | some patients I’ve seen, they were good. They accept here. They had no problem as long we explain to them. | |
| Clinic Pharmacy assistant: Pharmacy Assistant since 2019; Line 133 | | in your training receive any specific sort of education around TB preventive therapy? Uhm, was it part of your curriculum that you...?  Interviewee: no, it wasn't there. But I learnt it during my internship. | |
| Clinic OM: Female, 40s; Professional nurse for 25 years; OM for 1 year; Line 135 | | Has anybody talked to you about TB preventive therapy or how have you learned about it?  Interviewee: No, we just read from the clinical guidelines.  Interviewer: From the guidelines?  Interviewee: Yes.  Interviewer: Okay, alright. So that's kind of...  Interviewee: Specifically. The workshop that we usually have are the one for the TB. Then you get… they will have some portion of preventive therapy. | |
| Hospital OM: Male, 30s; 5 years at this hospital; Line 139 | | Even since I was training, but uh over the last years - even when I was training - it had always been uhm - like [name] would put it - the poor step sister kind of thing. It was always neglected. And as a result a lot of seniors that I was - and am still learning from - were little bit not clear, unclear about it as well. Guidelines were even a little uhm ambiguous and difficult to follow. A lot of it was tied to Tuberculants can test and manage - for a long while - and still at many places it's not possible or available. So yes, uhm in short I’d say it has been a very much neglected part partly because uhm - well I think partly just because a lot of people didn’t have a proper experience and there wasn’t very great guideline so it was always kind of brushed over and brushed away. | |
| Hospital OM: Male, 30s; <5 years at this hospital; Line 142 | | I think we - I mean we've discussed it here obviously partly because we have people who are passionate about TB care and partly because of the research that we’re doing. You know, this is, this TB research has been discussed I'm sure for years and years until COVID hit and then… uhm so we've spoken about IPT and then TPT for a long time and it is in our guidelines. So we speak about it, you know every so often in formal trainings provided by - I don't know if I‘ve, I probably haven't done that many formal trainings around TB care, so yeah. | |
| Pharmacist: Female, 30s; at this hospital for 3 years; Line 146 | | so I have not received any uhm like formal training. So like no workshops or whatever. Uhm most of the TPT I learnt about was because I was working the ARV unit and you know because I work here I have to know the guidelines. And so in the guidelines it says "When the CD4 count is this much then you must give the TPT. If the initiating treatment", all of that. That's how I generally learnt about it. I remember there was - I think some guidelines that were passed... Not passed, that were shared. I think an updated guideline, but that's just been about it. Everything I know it's stuff that I’ve read or I’ve had to read because of the patient population that I deal with. | |
| Clinic OM: Female, 40s; Professional nurse; 6 years at this clinic; Line 130 | | preventive therapy is very important because if you’ve got one, one person in the household that has tested for TB then obviously we screen the contacts. Then if the contacts are negative, if you put those contacts on preventive therapy for that period of six months - especially the children - then there will be no other positive TB in that household. | |
| Hospital MO: Male, 30s; MO at this hospital 7 years; Line 152 | | as part of a quit TB project. Uhm where it was trying to look at some of the things that I think that we're discussing now, on how to actually role out preventative therapy what’s the best way to eliminate TB in our sub district and district. And uhm a big part of that was looking at uhm TPT or TB preventative therapy. Uhm, so yeah there've been I think journal club talks hm about it. Uhm so on Tuesdays we usually have journal club where we discuss topics. Uhm it’s usually going through new guidelines, proposed guidelines uhm and programmatic things. Uhm so we did go uh into but yeah, then COVID happened last year and sort of died out a bit. | |
| Characteristics of individuals: self-efficacy | | | |
| Hospital MO: Female, 30s; MO for 5 years. Family Medicine registrar; Line 272 | | And I think nurses don’t want to be the person who does the wrong thing, so they’d rather not do anything. | |
| Hospital MO: Male, 40s; 15 years at this hospital; Line 274 | | And so I think that there might, I think that people are nervous about making a call in fear of making a mistake. So that they feel like "Is this TB or is this just latent TB...?" - I mean... "Is this, can I give them TBT or am I nervous?" And I think I mean it takes experience and I think people are a bit nervous to make that call. Uhm someone like [name] who does a lot will be very capable and confident of doing [it]. | |
| Hospital MO: Male, 40s; 15 years at this hospital; Line 275 | | People don't want to make a mistake. They don't want to do the wrong thing. And so I think that's... That kind of may be actually holding people back a bit, yeah. | |
| Hospital MO: Female, 30s; MO for 15 years; Line 277 | | They feel disempowered to actually do something about it, so they're not gonna be able to - they're not gonna force them. They're not gonna put a lot of emphasis on them to come in. | |
| Hospital MO: Male, 30s; <5 years at this hospital; Line 281 | | I think the current IPT guidelines are relatively specific on who qualifies for IPT so it should be easy for me to ask about children under five or children five and under and family mem - well household contacts with HIV because essentially that’s who gets it. No one else gets it. When we’re looking at more broadly providing preventative therapy it’s going to be along the list of, of candidates obviously. Uhm so as things are currently, I should be able to ask that question. Uhm, but I do still think the responsibility can lie with the TB nurses. I mean I, they… just in terms of volume of work required by different cadres of staff | |
| Process | | | |
| Hospital MO: Male, 30s; <5 years at this hospital; Line 83 | | I think, again I think nurses are far better screening than doctors and I know the way our system works here, an intentional TB screen is supposed to happen for every patient. And I think it does happen actually quite well because it's part of the triage process. | |
| Clinic OM: Female, 40s; Professional nurse for 25 years; OM for 1 year; Line 191 | | I have a team of TB/HIV who were doing the other programs. And I could see the difference. It's because they are the professional nurses.  Interviewer: okay. They were respected.  Interviewee: yes.  Interviewee: and at least they are knowledgeable on what they are doing. | |
| Characteristics of systems | |  | |
| Clinic Pharmacy Assistant. Pharmacy Assistant since 2019; Line 34 | | I don't know what's wrong with males, but they don't…the guys they don't take it seriously, yeah.” | |
| Hospital MO: Male, 30s; MO at this hospital 7 years; Line 16 | | “Definitely improving capacity at community level, primary health care level. So improving capacity for the nurses, linking care…in with the already existing programs that are running at the clinics - whether its family planning, immunizations for children, antenatal visits for adults - trying to make it seem more accessible and easier for the people involved. Involving the community health care workers because it’s one thing [screening] everybody and being able to prescribe the therapy, but it’s one thing getting people to take it and stay on it…I think it's multifaceted. It has to do with different levels of care and different levels of reaching the patient where they are and contextualizing their care.” | |
| Pharmacist: Female, 30s; at this hospital for 3 years; Line 24 | | I think - the pregnant women will be easy because - well not all of them, but majority of them do go to the clinics for antenatal care. So it will be easy to screen them because they show up you know themselves. With the children, because they have to get like immunizations done and children you know these childhood diseases. Today they're fine, tomorrow they've got diarrhea. They are gonna go to a clinic at some point between birth and the age of 5. So it's easier to screen them. With the HIV patients, because of the medication they're on, the fact that we have to monitor them, you know drawing bloods and stuff, they do have to access health care services. So any patient that I think has an illness will be easier to screen because they will seek medical attention at some point. Unlike the average Joe who's pretty healthy and may live in a house with poor people that have TB, but because he doesn’t have any symptoms, he’s just sitting at home. So yeah, I think those who have any sort of illness will be easier to screen because they will access health care at some point and so you can always just ask them questions. | |
| Hospital MO: Male, 40s; 15 years at this hospital; Line 222 | | I think if that was made available I think that I would really invest in, in community health workers quite frankly and saying... And kind of training them and in good protocols that... So, so I mean it would be about the logistics, about getting them out to households. But also just the thought around systems for making sure we - the people who we're not sure about, we get them back you know. So I mean I think that that would... We'd probably have three groups of people. One would be the kids under 5 - and having a good approach towards them - and I think we'd probably want to mentor them and then make a decision about whether they needs X-rays or not. Second group would be the none - HIV negative people... 00:20:10-4  Interviewer: HIV... 00:20:10-1  Interviewee: and HIV negative people I think one could probably - with a relative [inaudible 00:20:14-4] if there were no symptoms give them [inaudible 00:20:17-0] and see what - how they respond. And then the third group I think that, with HIV one we'd probably need to uhm be a little bit more thoughtful about getting them X-rays and things like that. So, so one would have to put fairly good protocols in place. So I think I would, I would think - have a thoughtful protocol, but also then empower uhm the community health workers to get to people. And that, that one would need transport for. So that's the hardest bit is actually getting them out into the community and getting them to people's houses. | |
| Hospital MO: Male, 30s; 5 years at this hospital; Line 171 | | “[The] challenge accessing health care is a big one, it really is a big one. And we do have quite a good network of clinics. So it’s not, it’s not impossible. It's not out - completely out of reach to access clinics. But having said that I'd say that that would [have to] be an absolute - it would be a clinic-based remedy. This hospital based…I don't think there's any way..” | |
| OM, Female, 40s; Professional nurse >10 years, OM for 1 year; Line 220 | | It’s when it comes to resources really it's a challenge, it's a big, big challenge. Especially on supplies, especially on transport - or personnel. | |
